# Artemisinin Alleviates Cerebral Ischemia/Reperfusion Injury via Regulation of the Forkhead Transcription Factor O1 Signaling Pathway

**DOI:** 10.1155/2022/7824436

**Published:** 2022-04-05

**Authors:** Xiaogang Yang, Ke Wu

**Affiliations:** ^1^Department of Neurosurgery, Affiliated Hospital of Yan'an University, Yan'an 716000, Shanxi Province, China; ^2^Department of Neurosurgery, Xichang People's Hospital, Xichang 615000, Sichuan Province, China

## Abstract

The effect and mechanism of artemisinin therapy on cerebral ischemia-reperfusion injury (CIRI) was analyzed in this work. 100 healthy male C57BL/6 mice were selected and randomly divided into the sham group (no treatment), CIRI model group (IR), IR + artemisinin posttreatment group (IR + Arte), EX527 + IR group (EX527 + IR), and EX527 + IR + artemisinin posttreatment group (EX527 + IR + Arte), with 20 mice in each group. The cerebral infarct volumes of mice in different groups were measured by the 2,3,5-triphenyltetrazolium chloride (TTC) staining method. The neurological function scores and oxidative stress levels of mice in different groups were measured and compared. In addition, the expressions of silent information regulator 1 (SIRT1), forkhead transcription factor O1 (FOXO1), and p53 protein in brain tissue were detected. The results showed that the contents of reactive oxygen species (ROS) and malondialdehyde (MDA) in the EX527 + IR group and EX527 + IR + Arte group were significantly higher than those in the IR + Arte group (*P* < 0.05). The expressions of SIRT1 protein in the brain tissue of the IR group and EX527 + IR group were much lower than that of the sham group (*P* < 0.01); compared with the IR + Arte group, the expression of the X527 + IR group in the brain tissue was greatly reduced (*P* < 0.05). The expression levels of FOXO1 protein and p53 protein in the brain tissue of mice in the IR group and EX527 + IR group were higher than those in the sham group (*P* < 0.01). It was concluded that artemisinin treatment can reduce oxidative stress damage and alleviate CIRI through the SIRT1/FOXO1 signaling pathway, thereby achieving neuroprotective effects.

## 1. Introduction

Ischemic stroke is a sudden and acute disorder of cerebral blood circulation, which has a high morbidity, fatality, and disability rate [1]. Among the current treatment methods for ischemic stroke, thrombolysis with recombinant tissue-type plasminogen activator and mechanical recanalization to establish cerebral blood flow reperfusion are the best treatment options, but it is easy to cause cerebral ischemia-reperfusion injury (CIRI) [2]. CIRI is an irreversible process, which is related to factors such as mitochondrial dysfunction, oxidative stress, apoptosis, inflammation, and ion imbalance in the body [3]. Artemisinin is an extract from the Compositae plant *Artemisia annua*. Current research results show that artemisinin has immunomodulatory, anti-inflammatory, antiapoptotic, and antifibrosis effects [4, 5]. Artemisinin can effectively reduce the myocardial CIRI through antioxidant and scavenging oxygen free radicals [6]. However, there are currently few studies on artemisinin posttreatment on brain protection, and its mechanism is not yet known [7].

Silent information regulator 1 (SIRT1) is a histone deacetylase that relies on nicotinamide adenine dinucleotide (NAD^+^), and it participates in cell proliferation, senescence, apoptosis, oxidative stress, and inflammatory reactions by cooperating with various factors such as forkhead transcription factor O1 (FOXO1) and nuclear transcription factor-*κ*B (NF-*κ*B) [8, 9]. It has been reported that the SIRT1/FOXO1 signaling pathway has a cerebral protective effect in a variety of brain diseases such as CIRI and subarachnoid hemorrhage [10]. Curcumin can reduce myocardial CIRI and large CIRI by activating the SIRT1/FOXO1 signaling pathway [11]. Chinese researchers Zhang et al. [12] pointed out that artemisinin has a certain effect on rat myocardial CIRI, but there is no report on the effect of artemisinin posttreatment on large CIRI and the specific mechanism.

## 2. Materials and Methods

### 2.1. Experimental Reagents

The experimental reagents included the malondialdehyde (MDA) kit (Nanjing Jiancheng Bioengineering Institute), superoxide dismutase (SOD) kit (Nanjing Jiancheng Bioengineering Institute), glutathione peroxidase (GSH-Px) kit (Nanjing Jiancheng Bioengineering Institute), MitoCheck Complex I Activity Assay Kit (Cayman Islands, USA), 2,3,5-triphenyltetrazolium chloride (TTC, Beijing Soleibo Biotechnology Co., Ltd.), paraformaldehyde (Hebei Tianda Chemical Co., Ltd.), and dihydroethidium (DHE).

### 2.2. Experimental Animals and Their Grouping

100 healthy male C57BL/6 mice aged 7-8 weeks and weighing 20–25 g were selected in this study; 5 mice per cage were housed in specified pathogen free (SPF) laboratory, with the room temperature of 25°C, relative humidity of about 55%, under 12-hour light conditions, and free drinking and eating. After 2 weeks of adaptive rearing, all mice were randomly divided into 5 groups: sham group (sham, no treatment), ischemia-reperfusion (IR) group (CIRI model), IR + artemisinin posttreatment group (IR + Arte), EX527 + IR group (EX527 + IR), and EX527 + IR + artemisinin posttreatment group (EX527 + IR + Arte), with 20 animals in each group. The details of time and route of administration are given in Table 1. All animal procedures in this experiment were approved by the Experimental Animal Management Committee, and the experimental methods were carried out in accordance with the approved guidelines.

### 2.3. Establishment of the Mice CIRI Model and Treatment Methods of Mice in Different Groups

A mouse middle cerebral artery occlusion model (MACO) [13] was used to establish a CIRI model, which was modified on this basis. During the operation, the mice were anesthetized with a mixture of oxygen and isoflurane, the neck and chest hair were removed, and the mice were fixed on the operating table in a supine position. A heating pad was adopted to maintain the rectal body temperature of mice at 37 ± 1°C. After routine disinfection of the head and neck, the anterior median incision of the neck was bluntly separated under a microscope to fully expose the right common carotid artery and external carotid artery of the mice. A 6-0 monofilament nylon thread with a length of 11 mm and poly-L-lysine-coated was inserted into the internal carotid artery through the common carotid artery to block the blood flow of the middle cerebral artery, induce ischemia for 30 minutes, and remove the thread plug for reperfusion. After the operation, the mice were resuscitated on a constant temperature plate at 37°C and then returned to room temperature for 24 hours.

Artemisinin was first completely dissolved in absolute ethanol and then diluted to normal saline at a volume ratio of 1 : 9. The mice except the sham group were injected intraperitoneally with artemisinin 10 minutes before brain tissue reperfusion. The dosage was 25 mg/kg. Before model construction, mice in the EX527 + IR and EX527 + IR + Arte groups were intraperitoneally injected with EX527 at a dose of 5 mg/kg, once every two days, for a total of 3 times.

### 2.4. Measurement of Neurological Function Score and Cerebral Infarct Volume

After 24 hours of reperfusion, mice in different groups were scored by three experimenters with unclear information about grouping, surgery, and dosage of mice individually with reference to Liang et al. [14] method mechanical energy neurological function score, and the average value was calculated and recorded. The normal state was rated as 0; the left front paw of the mouse could not be fully extended was rated as 1; the mouse turned to the left when awake was rated as 2 points; the mouse still had left side dumping in the resting state was rated as 3 points; and the mice were unable to walk or roll was rated as 4 points.

24 hours after reperfusion, pentobarbital sodium was used for anesthesia, the skin was cut along the middle of the mouse neck, the left mouse carotid artery was separated, the brain tissue was completely separated, weighed and sliced, and the thickness was about 2 mm. 2% 2,3,5-triphenyltetrazolium chloride (TTC) dye solution (Sigma-Aldrich, USA) was added for 30 min at 37°C under dark conditions, and 4% paraformaldehyde (Hebei Tianda Chemical Co., Ltd.) was added overnight. Then, the photos were taken. Red indicated normal brain tissue, and unstained indicated brain tissue in the infarct area. The infarct volume of mice in different groups was calculated.

### 2.5. Detection on Oxidative Stress Level, Mitochondrial Complex I (MCI), and ROS Activity

The malondialdehyde (MDA) kit (Nanjing Jiancheng Institute of Bioengineering), superoxide dismutase (SOD) kit (Nanjing Jiancheng Institute of Bioengineering), and glutathione peroxidase (GSH-Px) kit (Nanjing Jiancheng Institute of Bioengineering) were used to detect MDA, SOD, and GSH-Px contents in mouse brain tissues of different groups. The MitoCheck Complex I Activity Assay Kit (Cayman, USA) was used to detect the activity of MCI in the main member of the reactive oxygen species family in the brain tissue of different groups of mice.

The brain tissue was embedded with optimum cutting temperature compound (OCT) glue and frozen at −80°C for 1 min. After taking it out, the tissue was wrapped in tin foil and stored in the refrigerator at −80°C for later use. The tissue was cut into slices of about 10 *μ*m using a cryostat (Meikang, Germany), added with 10 mol/L dihydroethidium (DHE), and then treated at 37°C for 30 minutes. Next, it was photographed with a confocal microscope (Olympus, Japan) to analyze the fluorescence intensity with the Image Pro Plus software.

### 2.6. Western Blot (WB) to Detect Brain Tissue Protein Content

The tissue was rinsed with precooled phosphate buffer saline (PBS) and put into a homogenizer, added with 500 *μ*L of protein lysis solution for grinding, and fully lysed to extract total protein. The protein content was determined by the bicinchoninic acid (BCA) method. The sodium dodecyl sulfate polyacrylamide gel electrophoresis (SDS-PAGE) was adopted to separate the cellular proteins; the protein was transferred to polyvinylidene fluoride (PVDF) membrane by the wet transfer method and then sealed with 5% skimmed milk. After adding with primary antibodies diluted 1 : 1000, the tissue was incubated overnight at 4°C, thoroughly washed 3 times with Tris-buffered saline Tween (TBST), added with horseradish peroxidase-conjugated secondary antibody, and then incubated at room temperature for 2 hours. After it was thoroughly washed 3 times with TBST, the tissue was added with color-developing solution and automatically exposed on the developing instrument to take pictures for grayscale scanning. Finally, the protein expression level was analyzed according to the gray value.

### 2.7. Statistical Analysis

SPSS 19.0 was used for data statistics and analysis. Measurement data were expressed in the form of mean ± standard deviation ($\bar{x}$ ± *s*), and counting data were displayed in the form of percentage (%). Measurement data that obeyed the normal distribution were expressed by the *t*-test, or otherwise, they were expressed by Wilcoxon. In the test, one-way analysis of variance was used for comparison between groups, and *P* < 0.05 indicated that the difference was statistically significant.

## 3. Results

### 3.1. Comparison on Neurological Function Score Results in Different Groups of Mice

**Figure 1:**
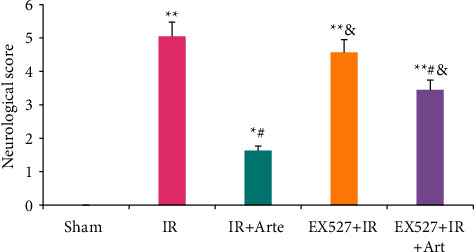
Comparison on neurological function score results in different groups of mice. ^*∗*^^, #, &^Difference was statistically great compared with the sham group, IR group, and IR + Arte group, respectively (*P* < 0.05). ^*∗∗*^Statistically extremely great difference in contrast to the sham group (*P* < 0.01).

The neurological function scores of mice in different groups were compared, as shown in Figure 1. The neurological function scores of mice in the IR group, EX527 + IR + Arte group, and EX527 + IR group were much higher than those in the sham group (*P* < 0.01), and compared with the sham group, there was a statistical difference in the IR + Arte group (*P* < 0.05). The neurological function scores of the mice in the IR + Arte group and EX527 + IR + Arte group were statistically different from those in the IR group (*P* < 0.05), and those in the EX527 + IR group and EX527 + IR + Arte group were visibly greater than the score in the IR + Arte group (*P* < 0.05).

### 3.2. Comparison on Cerebral Infarct Volume in Different Groups of Mice

**Figure 2:**
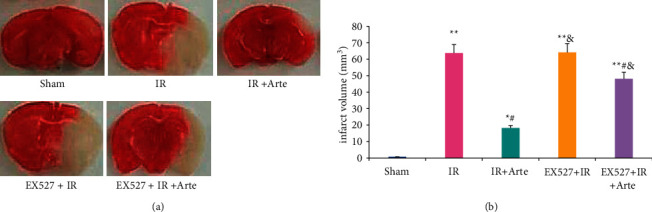
Comparison on cerebral infarct volume in different groups of mice. (a) TTC staining results. (b) The comparison of cerebral infarct volume. ^*∗*^^, #, &^Difference was statistically great compared with the sham group, IR group, and IR + Arte group, respectively (*P* < 0.05). ^*∗∗*^Statistically extremely great difference in contrast to the sham group (*P* < 0.01).

The results of TTC staining in the brain tissue of mice in different groups are shown in Figure 2(a). As it was shown, most of the area of TTC in the brain tissue of the IR group was unstained, and the TTC staining in the sham group was basically red. The cerebral infarct volume of mice in the IR group, EX527 + IR + Arte group, and EX527 + IR group was extremely obviously larger than that of the sham group (*P* < 0.01), and there was a statistical difference between the IR + Arte group and the sham group (*P* < 0.05). The cerebral infarct volume of mice in the IR + Arte group and EX527 + IR + Arte group was greatly different from that of the IR group (*P* < 0.05) and that in the EX527 + IR group and EX527 + IR + Arte group was dramatically larger than the IR + Arte group (*P* < 0.05) (Figure 2(b)).

### 3.3. Analysis of ROS Level and Oxidative Stress Level in Brain Tissue of Mice with Acute CIRI

**Figure 3:**
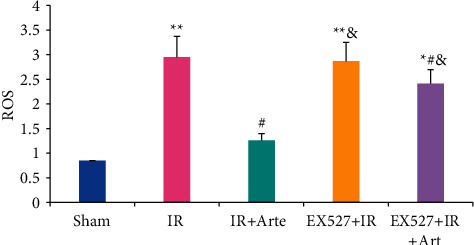
Comparison on ROS levels in brain tissue of mice with acute CIRI. ^*∗*^^, #, &^Difference was statistically great compared with the sham group, IR group, and IR + Arte group, respectively (*P* < 0.05). ^*∗∗*^Statistically extremely great difference in contrast to the sham group (*P* < 0.01).

The ROS levels in the brain tissues of mice in different groups were detected and analyzed, and the results are shown in Figure 3. The ROS levels in the brain tissues of mice in the IR group, EX527 + IR + Arte group, and EX527 + IR group were much higher than those in the sham group (*P* < 0.01). Compared with the IR group, the ROS levels of the mice in the IR + Arte group and EX527 + IR + Arte group were statistically different (*P* < 0.05) and that in the mice in the EX527 + IR group and EX527 + IR + Arte group were remarkably higher in contrast to the IR + Arte group (*P* < 0.05).

**Figure 4:**
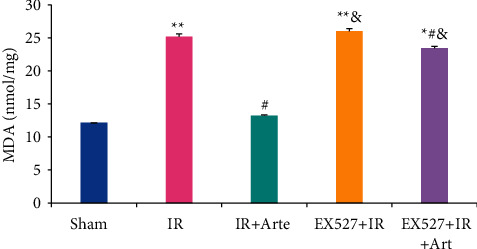
Comparison on MDA contents in brain tissue of mice with acute CIRI. ^*∗*^^, #, &^Difference was statistically great compared with the sham group, IR group, and IR + Arte group, respectively (*P* < 0.05). ^*∗∗*^Statistically extremely great difference in contrast to the sham group (*P* < 0.01).

The MDA contents in the brain tissues of mice in different groups were detected and analyzed, and the results are shown in Figure 4. The MDA content in the brain tissues of mice in the IR group, EX527 + IR + Arte group, and EX527 + IR group was much higher than those in the sham group, showing extremely statistical differences (*P* < 0.01). Compared with the IR group, the MDA contents of the mice in the IR + Arte group and EX527 + IR + Arte group were statistically different (*P* < 0.05) and those in the mice in the EX527 + IR group and EX527 + IR + Arte group were remarkably higher in contrast to the IR + Arte group (*P* < 0.05).

**Figure 5:**
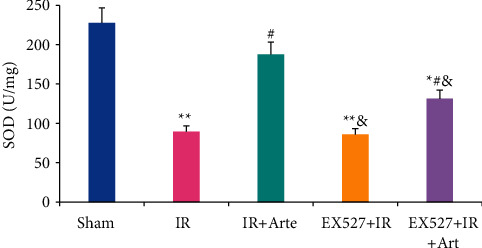
Comparison on SOD contents in brain tissue of mice with acute CIRI. ^*∗*^^, #, &^Difference was statistically great compared with the sham group, IR group, and IR + Arte group, respectively (*P* < 0.05). ^*∗∗*^Statistically extremely great difference in contrast to the sham group (*P* < 0.01).

The SOD contents in the brain tissues of mice in different groups were detected and analyzed, and the results are shown in Figure 5. The SOD contents in the brain tissues of mice in the IR group, EX527 + IR + Arte group, and EX527 + IR group were much lower than those in the sham group, showing extremely statistical differences (*P* < 0.01). Compared with the IR group, the SOD contents of the mice in the IR + Arte group and EX527 + IR + Arte group were statistically different (*P* < 0.05) and those in the mice in the EX527 + IR group and EX527 + IR + Arte group were remarkably lower in contrast to the IR + Arte group (*P* < 0.05).

**Figure 6:**
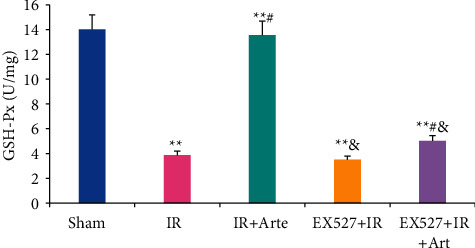
Comparison on GSH-Px contents in brain tissue of mice with acute CIRI. ^*∗*^^, #, &^Difference was statistically great compared with the sham group, IR group, and IR + Arte group, respectively (*P* < 0.05). ^*∗∗*^Statistically extremely great difference in contrast to the sham group (*P* < 0.01).

The GSH-Px contents in the brain tissues of mice in different groups were detected and analyzed, and the results are shown in Figure 6. The GSH-Px contents in the brain tissues of mice in the IR group, EX527 + IR + Arte group, and EX527 + IR group were much lower than those in the sham group, showing extremely statistical differences (*P* < 0.01). Compared with the IR group, the GSH-Px contents of the mice in the IR + Arte group and EX527 + IR + Arte group were statistically different (*P* < 0.05) and those in the mice in the EX527 + IR group and EX527 + IR + Arte group were remarkably lower in contrast to the IR + Arte group (*P* < 0.05).

### 3.4. Analysis on Protein Expression Related to the SIRT1/FOXO1 Signaling Pathway in Different Groups of Mice

**Figure 7:**
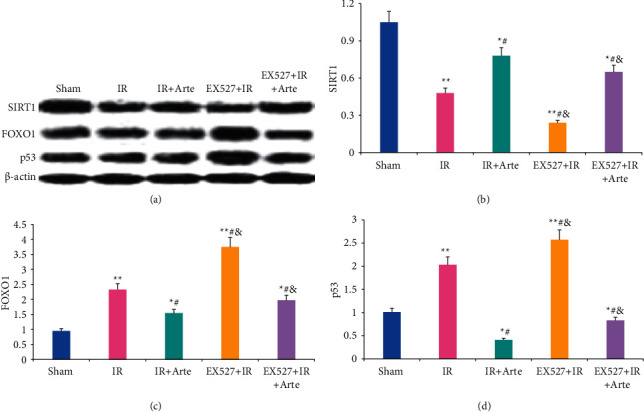
Analysis on protein expression related to the SIRT1/FOXO1 signaling pathway in different groups of mice. (a) The WB band. (b)–(d) The comparisons of SIRT1, FOXO1, and p53 expression levels. ^*∗*^^, #, &^Difference was statistically great compared with the sham group, IR group, and IR + Arte group, respectively (*P* < 0.05). ^*∗∗*^Statistically extremely great difference in contrast to the sham group (*P* < 0.01).

The SIRT1, FOXO1, and p53 protein expressions in the SIRT1/FOXO1 signaling pathway in the brain tissue of different groups of mice were analyzed, as shown in Figure 7. The expression of SIRT1 protein in the brain tissue of the IR group and EX527 + IR group was greatly lower than that of the sham group (*P* < 0.01), that in the IR + Arte and EX527 + IR + Arte groups was statistically significant in contrast to that in the sham group (*P* < 0.05), that in the IR + Arte group/EX527 + IR + Arte group and the EX527 + IR + Arte group was greatly different from that of the IR group (*P* < 0.05), that in the EX527 + IR group was lower visibly compared with that in the IR + Arte group (*P* < 0.05), and that in the brain tissue of the EX527 + IR + Arte group was lower greatly than that of the IR + Arte group (*P* < 0.05). The expressions of FOXO1 protein and p53 protein in the brain tissue of mice in the IR group and EX527 + IR group were observably higher than the expression in the sham group (*P* < 0.01), those in the brain tissue of the mice in the IR + Arte and EX527 + IR + Arte groups were statistically different from the sham group (*P* < 0.05), those in the IR + Arte group, EX527 + IR + Arte group, and EX527 + IR + Arte group were extremely different from those of the IR group (*P* < 0.05), those in the EX527 + IR group was higher than that in IR + Arte group (*P* < 0.05), and those in the brain tissue of the EX52 + IR + Arte group was significantly higher than that of the IR + Arte group (*P* < 0.05).

### 3.5. Activity Analysis on MCI in Rat Brain Tissue

**Figure 8:**
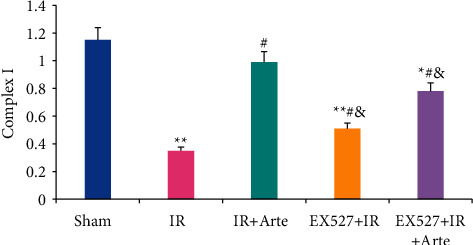
Comparison of activity on MCI in rat brain tissue. ^*∗*^^, #, &^Difference was statistically great compared with the sham group, IR group, and IR + Arte group, respectively (*P* < 0.05). ^*∗∗*^Statistically extremely great difference in contrast to the sham group (*P* < 0.01).

The activity of MCI in the brain tissue of mice in different groups was analyzed, as shown in Figure 8. The activity of MCI in the brain tissue of mice in the IR group and EX527 + IR group was extremely lower than that in the sham group (*P* < 0.01) and that in EX527 + IR + Arte group was significantly different from that in the sham group (*P* < 0.05); it in the IR + Arte group, EX527 + IR + Arte group, and EX527 + IR + Arte group was greatly different from that of the IR group (*P* < 0.05), and the activity of MCI in the EX527 + IR and EX527 + IR + Arte groups was lower in contrast to the IR + Arte group (*P* < 0.05).

## 4. Discussion

Oxidative stress can promote the occurrence and development of CIRI by inducing cell apoptosis and promoting inflammation [15, 16], and it plays an important role in CIRI. Studies have pointed out that inhibiting oxidative stress can effectively improve the blood-brain barrier caused by CIRI [17]. Ischemia-reperfusion will induce a large amount of ROS in the body, which will further initiate the process of inflammation and apoptosis, leading to neuronal cell death and aggravation of penumbra area damage [18]. Artemisinin has anti-inflammatory, antiapoptotic, and antioxidant effects [19] and is an antimalarial drug recognized by the World Health Organization. Current research results show that artemisinin can reduce myocardial fibrosis by downregulating the expression of transforming growth factor *β*1 protein, inhibit ventricular remodeling after myocardial infarction, and ultimately achieve a protective effect on the heart [20]. Franke et al. [21] pointed out that artemisinin can improve myocardial ischemia-reperfusion injury through an antioxidant mechanism. The results of this study showed that the levels of ROS and MDA in the brain tissue of mice in the IR group, EX527 + IR + Arte group, and EX527 + IR group were extremely higher than those in the sham group (*P* < 0.01), and those in the EX527 + IR group and EX527 + IR + Arte group were higher than those in the IR + Arte group (*P* < 0.05). The contents of SOD and GSH-Px in the brain tissue of mice in the IR group, EX527 + IR + Arte group, and EX527 + IR group were much lower than those in the sham group (*P* < 0.01), those in the IR + Arte group and EX527 + IR + Arte group were different from those in the IR group (*P* < 0.05), and those in the EX527 + IR group and EX527 + IR + Arte group were lower compared with the IR + Arte group (*P* < 0.05). In addition, the contents of ROS and MDA in the brain tissue of the IR group of mice increased greatly after ischemia-reperfusion, while the contents of SOD and GSH-Px decreased obviously. It shows that obvious oxidative stress damage has occurred after ischemia and reperfusion. After treatment with artemisinin, the content of ROS and MDA decreased, while the content of SOD and GSH-Px increased observably. It shows that artemisinin may improve oxidative stress damage after ischemia-reperfusion through antioxidant. Furthermore, the SIRT1 molecular-specific blocker EX527 was used to block the SIRT1/FOX1 signaling pathway. The results showed that the levels of ROS and MDA in the brain tissue of the mice in the EX527 + IR + Arte group were higher than those in the artemisinin-treated group, indicating that the SIRT1 signal is blocked, and the protective effect of artemisinin on the brain is weakened. MCI is an important member of the reactive oxygen species family, and it also plays an important role in the production of reactive oxygen species after cerebral ischemia and reperfusion [22]. The results in this study found that the activity of CMI in the brain tissue of mice in the IR group and EX527 + IR group was much lower than that in the sham group (*P* < 0.01), that in the EX527 + IR + Arte group was lower compared with the sham group with a statistical difference (*P* < 0.05), and that in EX527 + IR and EX527 + IR + Arte groups was lower in contrast to the IR + Arte group (*P* < 0.05). It suggests that the activity of MCI is significantly inhibited after CIRI, and it is increased greatly after artemisinin treatment, indicating that artemisinin can alleviate oxidative stress damage by activating MCI activity. After CIRI, a large number of free radicals will be produced in the body, and the activity of MCI will decrease, leading to damage to the nerves [23]. Artemisinin activates the activity of MCI to alleviate the damage of CIRI.

## 5. Conclusion

It explored the role of artemisinin in CIRI and its specific regulatory mechanism in the SIRT1/FOX1 signaling pathway. It was found that artemisinin can improve cerebral infarction volume and oxidative stress damage by regulating the SIRT1/FOX1 signaling pathway, relieving CIRI, and then achieving the neuroprotective effect. The results could provide a reference for the clinical treatment of CIRI. The disadvantage of this work was that only the oxidative stress factors in the SIRT1/FOX1 signaling pathway were detected, while the apoptosis-related factors in this pathway were not analyzed. In future works, it would further analyze whether oxidative stress factors in the SIRT1/FOX1 signaling pathway were involved in the protective effect of artemisinin on acute CIRI.

## Figures and Tables

**Table 1:** Time and route of administration.

Group	Administration time	Route of administration	Dose
Sham	10 minutes before brain tissue reperfusion	Intraperitoneal injection	25 mg/kg
IR	Before model construction	Intraperitoneal injection	5 mg/kg
IR + Arte	Before model construction	Intraperitoneal injection	5 mg/kg
EX527 + IR	Before model construction	Intraperitoneal injection	5 mg/kg
EX527 + IR + Arte	Before model construction	Intraperitoneal injection	5 mg/kg

## Data Availability

The data used to support the findings of this study are included within the article.
